# Environmental DNA method for estimating salamander distribution in headwater streams, and a comparison of water sampling methods

**DOI:** 10.1371/journal.pone.0176541

**Published:** 2017-05-17

**Authors:** Izumi Katano, Ken Harada, Hideyuki Doi, Rio Souma, Toshifumi Minamoto

**Affiliations:** 1 School of Human Science and Environment, University of Hyogo, 1-1-12 Shinzaike-Honcho, Himeji Japan; 2 Faculty of Science, Nara Women’s University, Kitauoyahigashi-machi, Nara, Japan; 3 Graduate School of Simulation Studies, University of Hyogo, 7-1-28 Minatojima-minamimachi, Chuo-ku, Kobe, Japan; 4 Graduate School of Human Science and Environment, University of Hyogo, 1-1-12 Shinzaike-Honcho, Himeji Japan; 5 Graduate School of Human Development and Environment, Kobe University, 3–11 Tsurukabuto, Nada-ku, Kobe, Japan; Leibniz-Institute of Freshwater Ecology and Inland Fisheries, GERMANY

## Abstract

Environmental DNA (eDNA) has recently been used for detecting the distribution of macroorganisms in various aquatic habitats. In this study, we applied an eDNA method to estimate the distribution of the Japanese clawed salamander, *Onychodactylus japonicus*, in headwater streams. Additionally, we compared the detection of eDNA and hand-capturing methods used for determining the distribution of *O*. *japonicus*. For eDNA detection, we designed a qPCR primer/probe set for *O*. *japonicus* using the 12S rRNA region. We detected the eDNA of *O*. *japonicus* at all sites (with the exception of one), where we also observed them by hand-capturing. Additionally, we detected eDNA at two sites where we were unable to observe individuals using the hand-capturing method. Moreover, we found that eDNA concentrations and detection rates of the two water sampling areas (stream surface and under stones) were not significantly different, although the eDNA concentration in the water under stones was more varied than that on the surface. We, therefore, conclude that eDNA methods could be used to determine the distribution of macroorganisms inhabiting headwater systems by using samples collected from the surface of the water.

## Introduction

Environmental DNA (eDNA) methods have been developed recently and are considered a new tool for investigating the distribution of aquatic macroorganisms [1, 2]. The use of eDNA methods for macroorganisms, particularly aquatic species, has increased recently. They have been applied to species inhabiting various aquatic habitats such as rivers, streams [3–7], lakes, ponds [8–13], and marine habitats [14–17].

eDNA methods in rivers and streams have been applied to several stream-inhabiting taxa: fish [18–20], crustaceans [21], mollusks [22], and amphibians [4, 5]. eDNA studies expand our knowledge about the use of eDNA methods to estimate the abundance or biomass of species [4, 9, 19, 20], and help to evaluate how eDNA travels downstream [22, 23]. Moreover, eDNA may be more useful for detecting burrowing species than traditional methods such as kicking-net sampling and hand-capturing, because it is difficult to locate hiding or burrowing individuals under stones [22].

The use of eDNA methods for aquatic species has three major merits: (1) reduced cost and working time for sampling [2]; (2) reduced disturbance impacts on habitats; (3) reduced safety risk for investigators during field surveys. In streams with strong currents and deep water, direct sampling of benthic species may sometimes be difficult and risky for researchers working in these conditions. For safety during fieldwork, eDNA methods may potentially be useful because only small water samples are necessary for detection. We should consider that eDNA concentrations and detection may differ between water at the surface and at the bottom or under stones, because many stream species are found under stones and not in the open water. However, this has yet to be explored.

In this study, we used eDNA methods to evaluate the distribution of the Japanese clawed salamander, *Onychodactylus japonicus*, which inhabits headwater streams at high altitudes. We compared the results of eDNA detection by quantitative real-time PCR (qPCR) with those of the traditional survey method, hand-capturing. The Japanese clawed salamander is an endangered species listed in some Japanese Prefectures’ Red Lists. For example, this species is listed under the Threatened (Vulnerable, VU) category (http://www.jpnrdb.com/index.html, 12 Sep. 2016) in Hyogo Prefecture, where our study area was located. Furthermore, we compared the eDNA concentrations and detection of two water sampling methods: by collecting samples on the surface of the water, and under stones where the species are found.

## Materials and methods

### Study species

*O*. *japonicus* is distributed in the mountains of Honshu and Shikoku islands [24]. The species breeds at stream heads, and hatched larvae grow in the mountain streams for about 2 years before metamorphosis [25]. The breeding season is estimated to occur from April to June. Hibernation occurs from November or December to April on land, e.g., under stones [24].

### Study site

**Fig 1:**
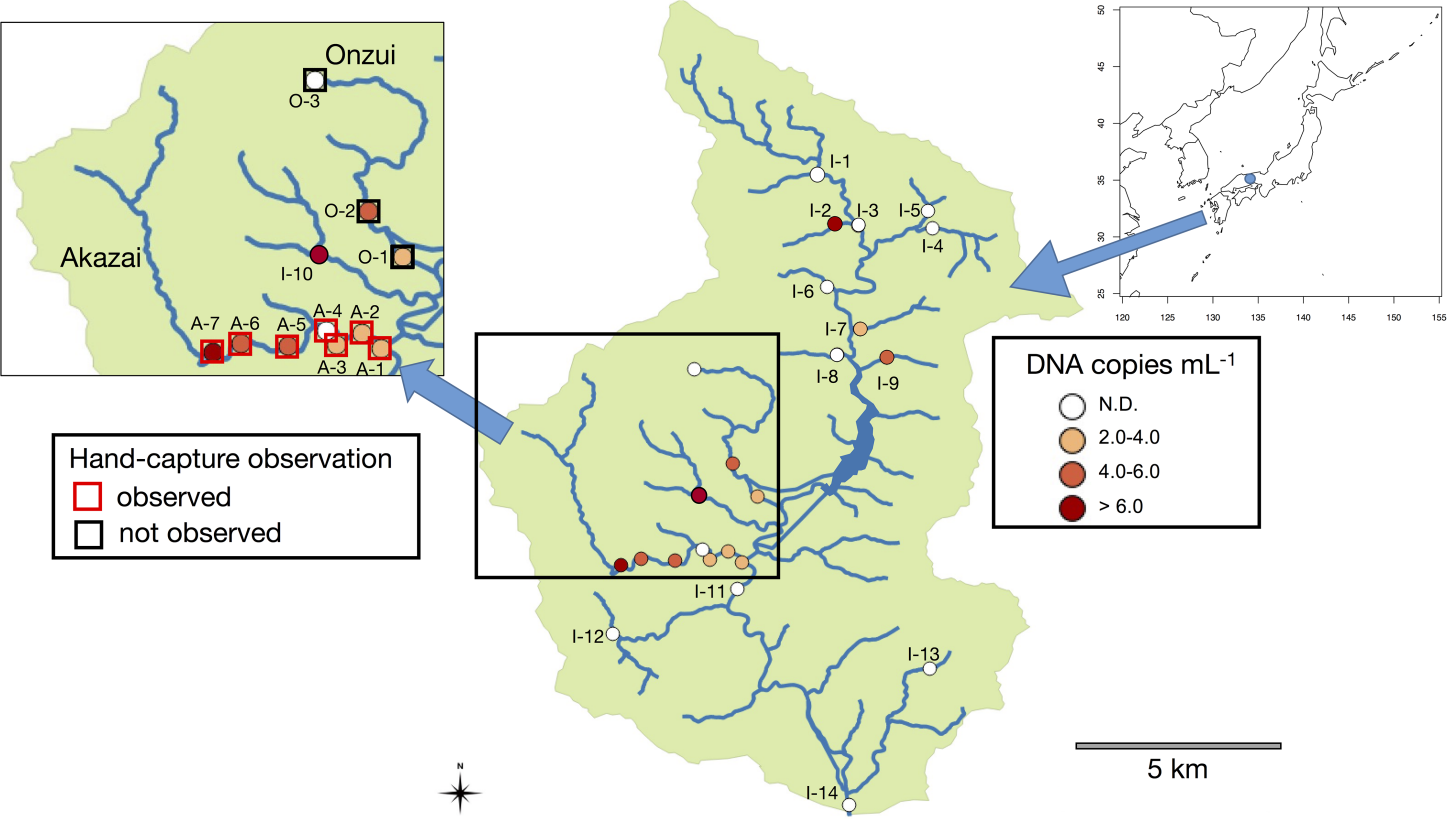
Map of survey sites and eDNA concentration of water on the surface of headwater streams. At the sites on the expanded map (A1-A7, O1-O3), we directly captured the species using the hand-capture method (Table 1). N. D. means eDNA was not-detected.

For eDNA sampling and hand-capture surveys, our study sites were located in the headwater tributaries of the Ibo River in Hyogo, Japan (Fig 1, Table 1). We recorded the location of each survey site using a GPS (eTrex30J, GARMIN, Olathe, KS, USA). Seven sites were in the Akazai River (A1-A7, numbered upstream), and three were in the Onzui River (O1-O3). The orders of the streams and width of the tributaries were 1–2 and 4–8 meters respectively. The water depth ranged from approximately 0–70 cm with a current of 0–50 cm s^-1^ (Harada et al. unpublished data). Additionally, there were 14 sites in the tributaries of the Ibo River (I1-I14). Stream orders of the tributaries of the Ibo River were 3–5. The river has a reservoir lake formed by the Hikihara dam (dam height: 66 m, watershed area: 57 km^2^, Fig 1). For the comparison of water sampling methods, we set up nine additional sites between A1 and A7 in the Akazai River (35°14′28.6″N 134°29′00.5″E). *O*. *japonicus* has not been observed in the area (I-1 to I-9) upstream of the Hikihara Reservoir by the local people for decades; thus, we assumed this species did not inhabit the area. No specific permits were required for the described field studies.

**Table 1:** The results of hand-capture surveys and eDNA detection (September and October) for *O*. *japonicus*.

Site	Name	Observation	eDNA Detected
A-1	Akazai	+	1/8
A-2	Akazai	+	3/8
A-3	Akazai	+	1/8
A-4	Akazai	+	0/8
A-5	Akazai	+	3/8
A-6	Akazai	+	4/8
A-7	Akazai		8/8
O-1	Onzui	–	2/8
O-2	Onzui	–	5/8
O-3	Onzui	–	0/8
I-1	Ibo	*	0/8
I-2	Ibo	*	3/8
I-3	Ibo	*	0/8
I-4	Ibo	*	0/8
I-5	Ibo	*	0/8
I-6	Ibo	*	0/8
I-7	Ibo	*	2/8
I-8	Ibo	*	0/8
I-9	Ibo	*	4/8
I-10	Ibo	not recorded	8/8
I-11	Ibo	not recorded	0/8
I-12	Ibo	not recorded	0/8
I-13	Ibo	not recorded	0/8
I-14	Ibo	not recorded	0/8

Observed, not observed by our study, and not observed by personal communication, are represented by +, -, and *, respectively.

The ratios in ‘eDNA Detected’ indicate positive samples / qPCR replicates.

### Field sampling for eDNA

**Fig 2:**
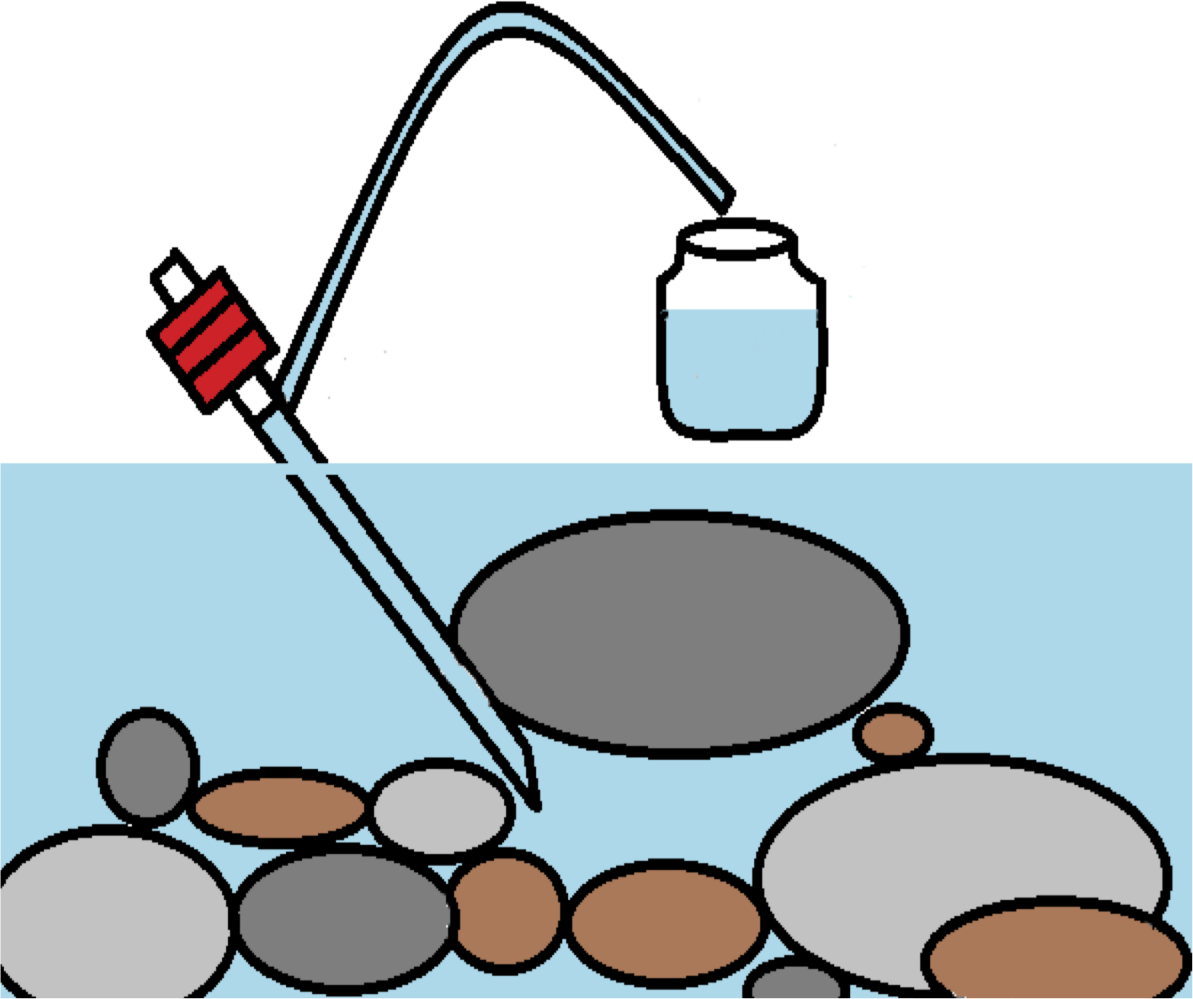
Illustration water sampling under stones.

For the distribution survey using eDNA, we collected a 1 L sample from the surface of the water, at the center of each stream in the study sites, using a DNA-free polypropylene bottle. Each bottle used for water collection was bleached with 0.6% hypochlorous acid and washed using DNA-free distilled water (DW). Comparison of the water sampling methods (at the surface and under stones) was conducted at an additional nine sites on the Akazai River. We collected 5 L of water on the surface at the center of the stream, and 1 L of water under stones, where we found individuals of our target species. We used a Kerosene siphon pump (40 cm length, diameter 1.3 cm, Pencopet, GJ-10, Trusco, Tokyo, Japan) to collect each water sample from under stones into the polypropylene bottle (Fig 2) just before our hand-capture survey of *O*. *japonicus*. We used the water collected from under stones for eDNA analysis only when we confirmed the presence of individuals at that site. We increased the volume of water collected at the surface to 5 L, because the suitable volume of water needed for eDNA sampling was unknown. In contrast, we were able to collect 1 L of water from under the stones before the pump began to siphon sand from the stream bottom.

Samples were stored in a cooler box with a ‘cooler blank’ [19, 26]. The ‘cooler blank’ contained 1 L DW, which we brought to the field, and it was treated identically to the other water sampling bottles except that it was not opened at the field sites. The bottles and siphon pumps used for water collection were bleached using 10% commercial bleach (*ca*. 0.6% sodium hypochlorite) and washed and rinsed twice with DW. The surveys were conducted in May (at the additional sites for sampling on surface/under stone), September, and October 2015 (for species distribution in the Ibo River watershed).

The collected water samples were vacuum-filtered through 47-mm GF/F glass filters (pore size 0.7 μm, GE Healthcare, Little Chalfont, UK). The filter was then wrapped in commercial aluminum foil and stored at -20°C before eDNA extraction. We incorporated the ‘equipment blank’ as a negative control (1 L DNA-free DW, which was filtered similarly after the filtration of the samples on each sampling day), and the ‘cooler blank’ was filtered at the same time. The DNA of these negative controls was tested along with the sample filters to identify any field preparation/transportation, filter equipment, or background contamination. The filters were then stored at -20°C until DNA extraction.

### DNA extraction from the filters

To extract DNA from the filters, we followed the methods described by Uchii et al.

[13]. We incubated each filter in a mixed buffer of 400 μL of Buffer AL (Qiagen, Hilden, Germany) and 40 μL of Proteinase K (Qiagen, Hilden, Germany), using a Salivette® tube (Sarstedt, Nümbrecht, Germany) at 56°C for 30 min. The Salivette tube with the filter was centrifuged at 3,500 × *g* for 5 min, and then we added 220 μL of the TE buffer (10 mM Tris-HCl and 1 mM EDTA, pH: 8.0) to the filter and centrifuged at 5,000 × *g* for 5 min. The dissolved DNA in the eluted solution was purified using a DNeasy Blood & Tissue Kit (Qiagen, Hilden, Germany) according to the manufacturer’s protocol. The final volume of the extracted sample was eluted in 100 μL of Buffer AE of the DNeasy Blood & Tissue Kit, and this was stored at -20°C until qPCR analysis.

### Primer-probe design for *O*. *japonicus*

To detect and quantify the DNA of *O*. *japonicus* using qPCR, we developed species-specific primers that amplify a 123-bp fragment of the 12S ribosome gene of mitochondrial DNA. The design of the primers and TaqMan® probe were as follows: Ony-j_12S_F (5′-TACTTGAAACCACGACCGCT-3′), Ony-j_12S_R (5'-CGCCAAGTCCTTTGAGTTTT-3′), Ony-j_12S_probe (5′-[FAM]-TCCGCCAGATTACTACGAGC-[TAMRA]-3′). The specificity of the primers and probe was tested with sequences of *Onychodactylus* and *Hynobius* species that are present in Japan, from the National Center for Biotechnology Information databases (NCBI, http://www.ncbi.nlm.nih.gov/): *Hynobius kimurae*, *H*. *dunni*, *H*. *abei*, *H*. *hidamontaus*, *H*. *okiensis*, *H*. *retardatus*, *H*. *stejnegeri*, *H*. *takedai*, *H*. *tsuensis*, *H*. *boulengeri*. In addition, *Onychodactylus* species that do not occur in Japan were tested: *O*. *fischeri* and *O*. *zhangyapingi*. The salamander species found in Japan were not detected during the *in silico* specificity screen, which was performed using Primer-BLAST (http://www.ncbi.nlm.nih.gov/tools/primer-blast/). In addition, we performed *in vivo* specificity tests using 100 pg per PCR reaction of the extracted DNA from *H*. *kimurae*, which was the only other salamander species inhabiting the study regions with *O*. *japonicus*.

### Quantitative real-time PCR

The eDNA was measured using a PikoReal™ qPCR system (Thermo Fisher Scientific, Waltham, MA, USA). Each PCR reaction mixture contained 900 nM of each primer (F, R), 125 nM of the TaqMan probe in a 1× PCR master mix (TaqMan Environmental Master Mix 2.0; Life Technologies), and 2 μL of the DNA solution. The total volume of each reaction mixture was 10 μL. The PCR conditions were as follows: 10 min at 95°C, 55 cycles of 15 s at 95°C, and 60 s at 60°C. There were eight replicates for each PCR sample. If any of these replicates yielded a positive value, the presence of *O*. *japonicus* DNA was assigned to it. Each real-time PCR assay included eight no template controls (NTC), and there were no amplifications from the negative controls. We set eight replications for the real-time PCR measurement as in the previous eDNA studies for detecting aquatic species [11, 17, 21]. The real-time PCR procedures were run according to the MIQE checklist ([27], see S1 File). We performed the PCR set up and real-time PCR in two separate rooms to avoid contamination.

The qPCR results were analyzed using PikoReal software ver. 2.2.248.601 (Thermo Fisher Scientific, Waltham, MA USA). A standard curve for the target was constructed using the dilution series of 10 000, 1000, 100, and 10 copies per PCR reaction. For the standard curve, we used the target DNA cloned into the plasmid. The R^2^ values of the standard curves ranged from 0.985 to 0.993 and the PCR efficiency from 78.45 to 95.65%. The concentration of DNA in the water collected (DNA copies mL^-1^) was calculated from the volume of filtered water. DNA copy numbers were evaluated including negative amplifications as zero. The limit of detection (LOD) of qPCR for eight replicates was one copy per reaction.

### PCR inhibition test

We used ‘Ct shift’ between samples with the same number of known target DNA copies as a measure of the relative degree of inhibition [28, 29]. Ct is defined as the number of cycles required for sufficient amplified PCR product to accumulate, so that it crosses a threshold recognized by the qPCR instrumentation. Ct is inversely related to starting quantity of target DNA in a reaction, and is used to calculate this quantity [30]. Presence of PCR inhibitors will shift (delay) the Ct for a given quantity of template DNA.

To test for inhibition in the DNA samples, we spiked the PCR template with 1 μL of a plasmid containing the cytochrome b gene of *Trachurus japonicus* (a marine fish not found in the sample area) (1.5 × 10^3^ copies), in place of 1 μL of DW. The primer and probe set was cited from Yamamoto et al. (2016) [17]: forward primer, 5′-CAGATATCGCAACCGCCTTT-3′; reverse primer, 5′-CCGATGTGA AGGTAAATGCAA A-3′; probe, 5′-FAM-TATGCACGCCAACGGCGC CT-TAMRA-3′. The presence of PCR inhibitors was evaluated as ΔCt (Ct_positive control_—Ct_sample_). A ΔCt ≥ 3 cycle can be considered evidence of inhibition [28].

### Direct sequence of PCR amplicon

To confirm primer specificity, we performed direct sequences of the qPCR amplicon in the Akazai and Onzui sites which were collected in the fall (*N* = 9, A1-A7, O1-O3, excluding A6 and O3, Table 1). The PCR amplicons were directly sequenced after treatment with ExoSAP-IT (USB Corporation, Cleveland, OH, USA). Sequences were determined by a commercial sequencing service (Eurofins Genomics, Tokyo, Japan).

### Hand-capture survey

To estimate *O*. *japonicus* distribution directly, we performed hand-capture surveys in the Akazai and Onzui rivers after the collection of water from the surface for eDNA testing (Table 1). We recorded the presence or absence of species by hand-capturing, and checked the habitats under stones at the sites within 20 min of water collection. Observation was limited to an area within approximately 20 m of the study sites. One person (K. Harada) performed the surveys.

### Statistical analyses

We calculated Cohen’s Kappa value [31] to compare the detection rates of *O*. *japonicus* distribution between hand-capturing and eDNA detection methods. The paired Welch’s t-test was performed to compare the DNA concentration and ΔCt value of water collected from the surface and under stones. We performed a Pearson’s correlation coefficient to determine the correlation between the water samples from the surface and under stones. The significance of all statistical data was set at α = 0.05. All statistical analyses and graphics were conducted with R version 3.2.3 [32] as well as IRR ver. 2.0.0 and ggplot2 ver. 2.0.0 for Cohen’s Kappa calculation. For the map graphics, we used map plotting in QGIS ver. 2.12, and the stream map data was obtained from the National Land Information Division, Ministry of Land, Infrastructure, Transport, and Tourism of Japan (http://nlftp.mlit.go.jp/ksj/). The regional map data was from "maps" package in R.

## Results

### Primers and probe testing

**Fig 3:**
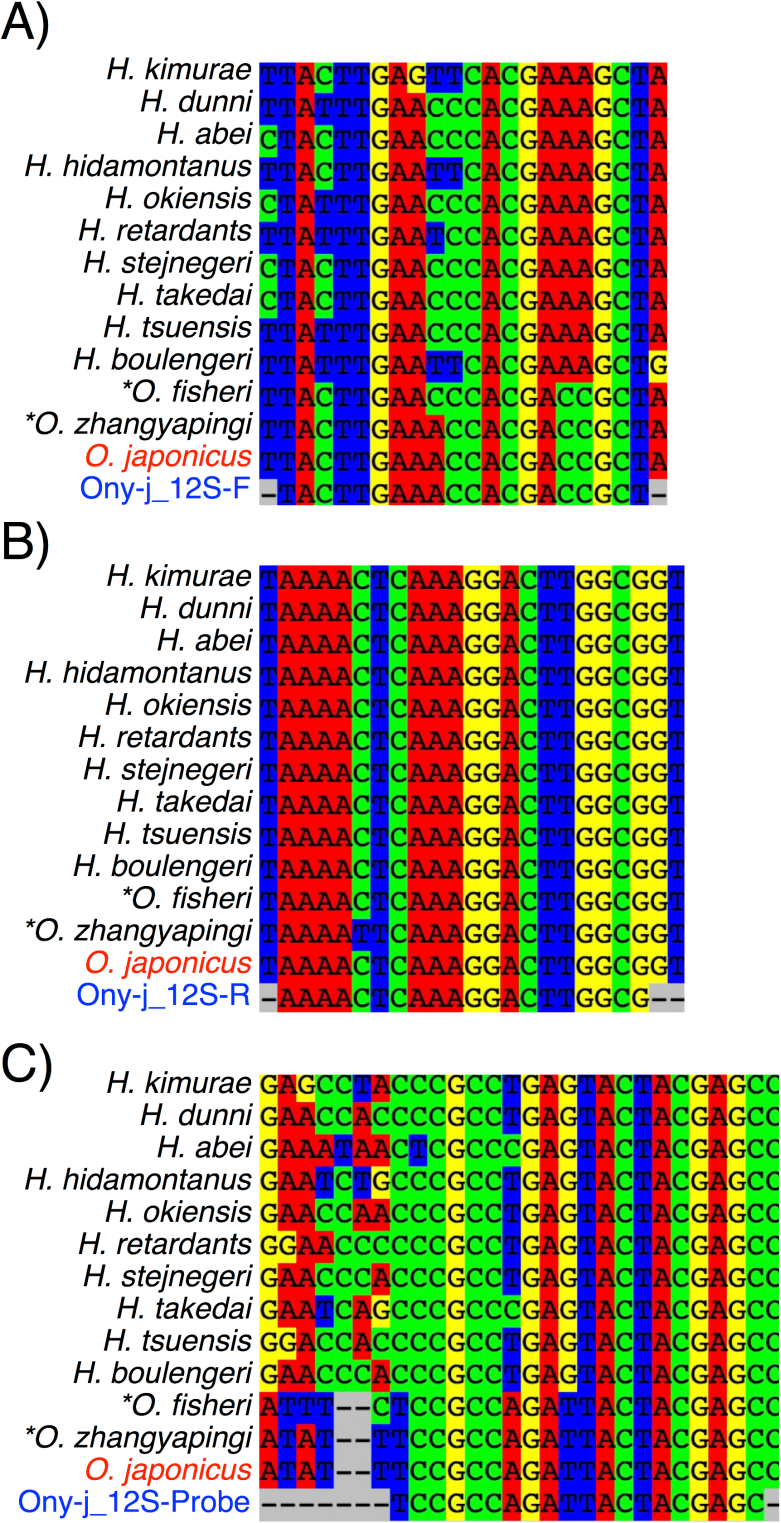
The designed PCR primer/probe set (Ony-j_12S_F (A), _R (B), and _Probe (C)) for *O*. *japonicus* in 12S rRNA and sequence information of *O*. *japonicus*, other *Onychodactylus* species found outside Japan (*), and *Hynobius* species found in Japan. Note the sequence of Ony-j_12S_R was a reverse complement.

We tested the matching of the qPCR primer/probe set for the DNA sequencing data of *O*. *japonicus* by comparing it with other non-target *Hynobius* and *Onychodactylus* species (Fig 3). All sequencing data were obtained from the NCBI database (https://www.ncbi.nlm.nih.gov).

The set did not detect any DNA of *Hynobius* species in Japan from the *in-silico* comparison. In our experiment, we confirmed that the DNA from *H*. *kimurae*, another salamander species inhabiting the study areas, was not amplified by the primers and probe. The Ony-j_12S_R was universal for all sequencings of *Hynobius* and *Onychodactylus* species, except *O*. *zhangyapingi*, but we did not detect any positives for the DNA of *H*. *kimurae* by the *in vivo* specificity tests using the extracted DNA. However, the primer/probe set possibly detected *Onychodactylus* species that were not present in Japan (*O*. *fischeri*). The DNA sequencing data of the qPCR amplicons confirmed that all the sequences belonged to *O*. *japonicus*.

### Spatial distribution of eDNA concentration

The spatial distribution of *O*. *japonicus* across the watershed was evaluated by mean eDNA concentration (Fig 1). By the PCR inhibitor test, ΔCt from internal controls were lower than 1.5 (see S1 Table), indicating the PCR inhibitors did not affect the qPCR results or cause non-detection of the DNA. The eDNA concentrations were remarkably higher in the streams around the Akazai and Onzui rivers. We detected eDNA from all the sites, except one, in the Akazai River where we found species by hand-capturing (Table 1). Cohen’s Kappa value for comparing the hand-capture and eDNA methods was 0.25, and not significantly different from the null model (z = 0.894, p = 0.371), indicating that the detectability of the study species was not significantly different between the hand-capture and the eDNA methods. Moreover, we detected eDNA from water upstream of the reservoir lake, where the distribution of *O*. *japonicus* was not observed previously.

### Comparing water sampling methods for eDNA

**Fig 4:**
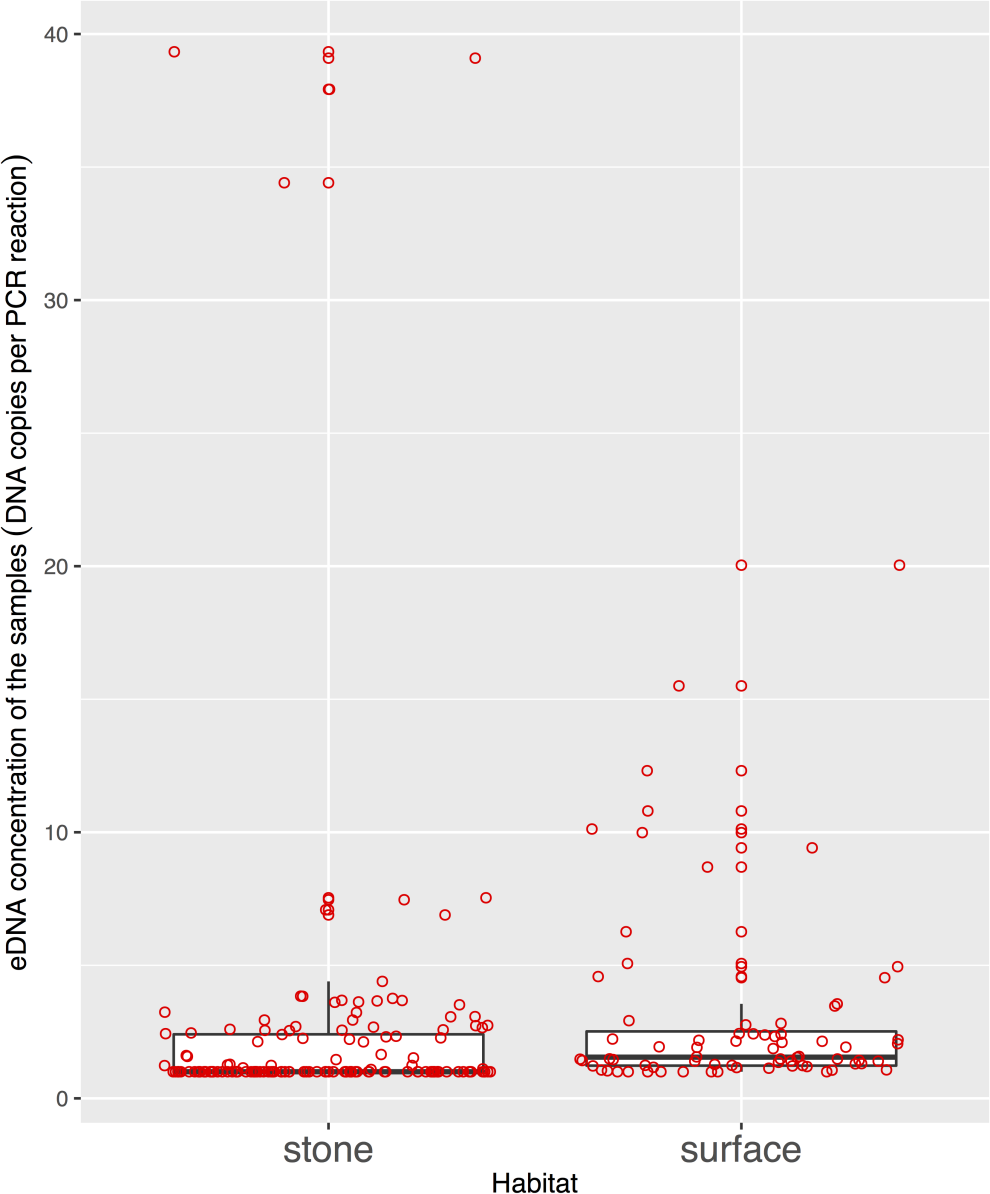
The eDNA concentrations (in PCR reactions) from water samples collected from the surface and under stones at sites in the Akazai River. The bold line in the box indicates the median value. The upper and lower limits of the box and the whisker plots indicate the first and third quartiles and ± 1.5 × interquartile ranges, respectively. The red dots represent each data point.

**Fig 5:**
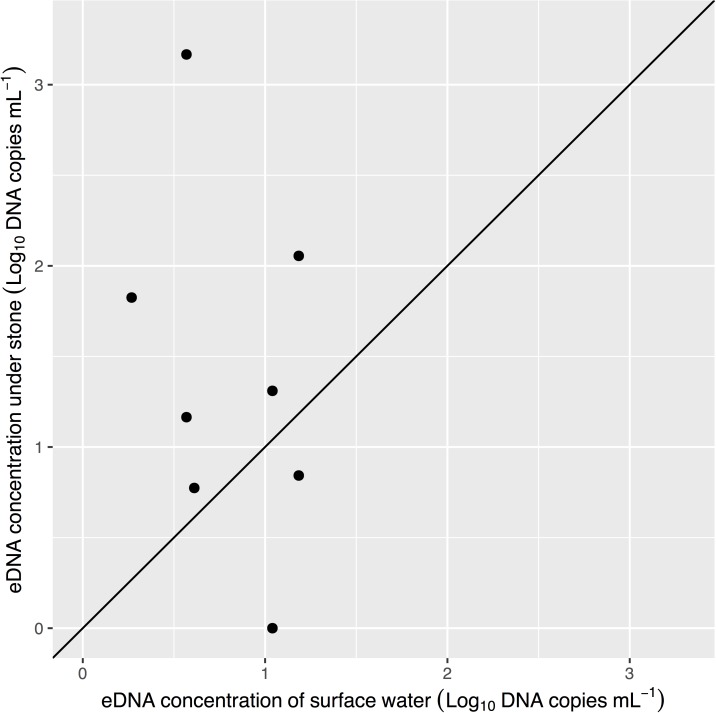
Comparison of mean eDNA concentration of the water samples collected from the surface and under stones at sites in the Akazai River. The line shows 1:1 correlation for the log_10_ concentrations.

**Fig 6:**
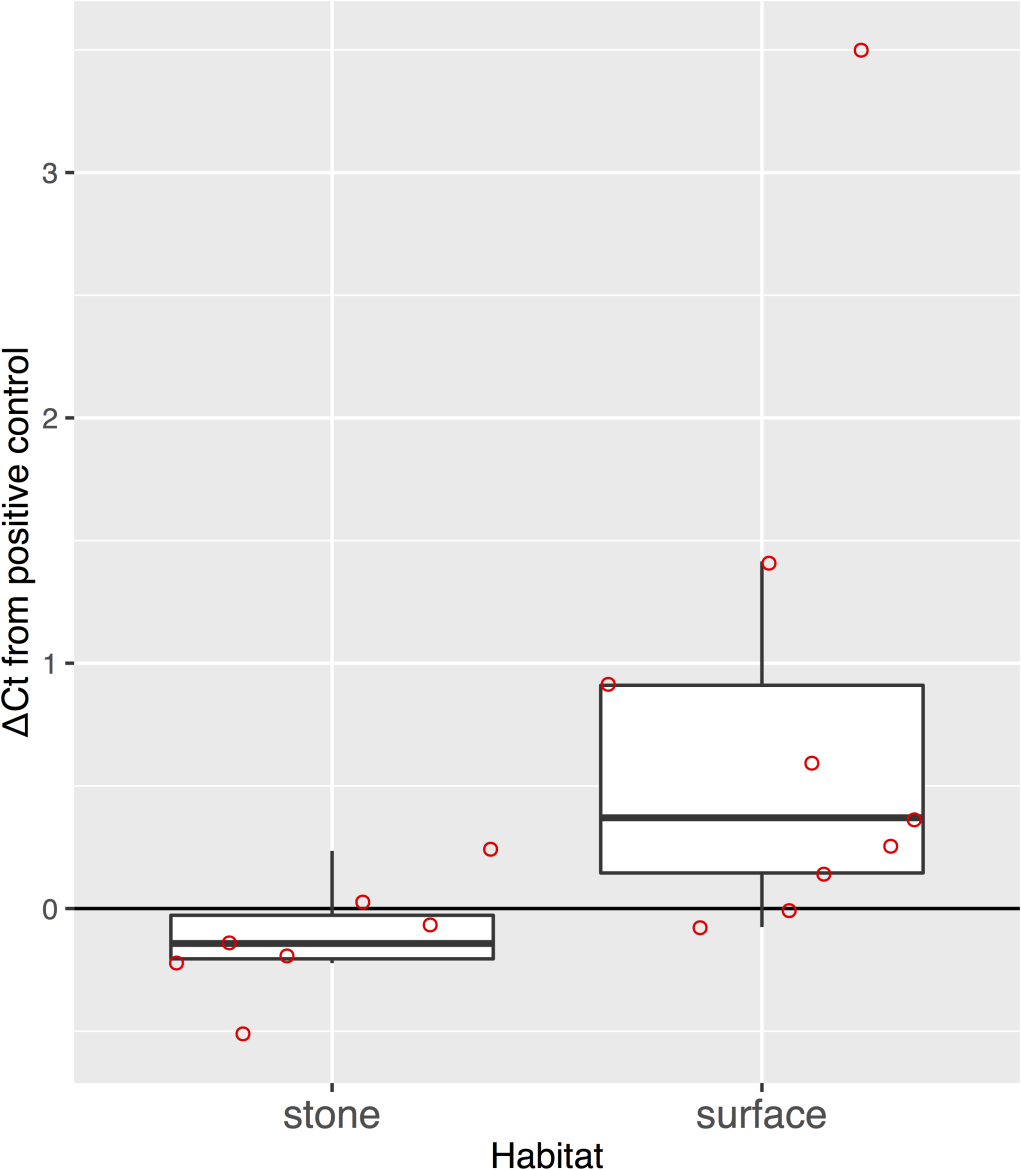
The mean ΔCt value from internal positive controls by the PCR inhibition test for the samples collected from the surface and under stones at sites in the Akazai River. The bold line in the box indicates the median value. The upper and lower limits of the box and the whisker plots indicate the first and third quartiles and ± 1.5 × interquartile ranges, respectively. The red dots represent each data point.

The eDNA concentration in the PCR reaction was not significantly different between water samples collected from the surface of the streams and under stones (Fig 4, paired Welch’s t-test, t = -0.527, df = 207, p = 0.600). In addition, eDNA concentration under the stones could be either higher or lower than that collected from the surface. In addition, there was no significant correlation between the concentrations of the two sampling methods (Pearson’s correlation coefficient, r = 0.338, p = 0.412). In a site where we detected the eDNA in the water at the surface, we could not detect any in the water under stones (Fig 5). The ΔCt from internal controls was lower than 3 (Fig 6), except for the surface water from one site, and the ΔCt values were significantly different between water samples collected from the surface of the streams and under stones (Fig 6, paired Welch’s t-test, t = –4.062, df = 41.4, p < 0.001). We found a high copy number with many negatives in the replicates in some samples, but assumed these were not false positives because we did not detect any false positives in the NTC and blank samples, including cooler and equipment blanks.

## Discussion

In most cases, we detected eDNA at sites where we also hand-captured *O*. *japonicus*; however, we also detected eDNA where we could not directly hand-capture. Thus, the eDNA method can evaluate the distribution of *O*. *japonicus* as effectively as classical observation methods such as the hand-capture method, while being more time- and cost-efficient. The eDNA method for detecting *O*. *japonicus* distribution may therefore be useful as a new monitoring tool for small salamanders in headwaters. It should be noted that the results for species detection with the hand-capture and eDNA methods were significantly different at site A4, where we observed species by hand-capture, but could not detect eDNA. Hence, the eDNA method may also produce false negatives when estimating species distribution. Such differences in sampling methods are generally observed [5, 19] and should be taken into account when using the eDNA method. The eDNA method is a non-invasive and effective method, due to the ease of collecting surface water. In addition, DNA identification allows identification of species, which are difficult to identify on the basis of their morphology. Furthermore, classical hand-capture methods allow us to obtain more information about the individuals, such as body mass and local habitats. The benefits and limitations of both methods should be considered when choosing the survey methods to apply to a targeted species.

The primer/probe set for *O*. *japonicus* can be used in streams in Japan, where no other *Onychodactylus* species inhabit. However, in other regions, *Onychodactylus* species are not distinguishable using the primer/probe set. Indeed, the taxonomy and phylogeny of *Onychodactylus* species is still under debate [33]. Yoshikawa [34] suggested that Japanese *Onychodactylus* (*O*. *japonicus*) would have four major haplotype clades across Japan, including this study region, where *O*. *japonicus* III-C was distributed. Thus, in this study, we may have detected *O*. *japonicus* III-C as *O*. *japonicus*. This primer/probe set can be used to detect *Onychodactylus* spp., but not each clade and species, indicating that we should use the primer/probe set cautiously in regions where multiple species of *Onychodactylus* are present. Nevertheless, such genus level-specific primer/probe sets may be useful for broad scale biological monitoring.

The eDNA of *O*. *japonicus* at the study sites may partly be from the upstream reaches. However, Pilliod *et al*. [4] measured the eDNA of the salamander, *Dicamptodon aterrimus*, and found the highest correlation coefficient between salamander density and eDNA concentration in a stream where the eDNA sample was collected at the survey site with consideration of the upstream/downstream distance from the survey site. Moreover, Deiner & Altermatt [22] suggested that the eDNA of zooplankton and shellfish traveled approximately 10 km downstream. In this study, we did not investigate the actual eDNA source at the site, but from the results of the study by Pilliod *et al*. [4] with a similar-sized salamander, we speculated that the eDNA from areas upstream did not largely affect the estimation of eDNA. In fact, we did not detect eDNA at a site (Site A-4) in the Akazai River where we detected the eDNA upstream.

We found no significant difference between the eDNA concentrations of water samples collected from the surface and under stones where individuals were found. In the water under stones, we generally found higher maximum concentrations of eDNA than that on the surface. This may be due to the individuals' condition or microhabitat structure, such as their body mass and water transparency in the cobbles. The salamander species has a burrowing habit in the cobbles, but for such species, the eDNA analysis using surface stream water is sufficient when comparing the eDNA from their microhabitats. Furthermore, in some sites where we detected eDNA from individuals, it did not remain in the microhabitat due to high water transparency. This may be because eDNA was not captured in the water sample despite individuals being observed. We assumed a greater effect of PCR inhibitors, such as humic acids from deposited litter, in the water samples from under stones than in those from the surface; however, PCR inhibition was not generally found there. The ΔCt values of surface water samples were significantly greater, probably due to greater amounts of PCR inhibitors found in the larger water volume (5 L). The results suggest that water sampling from the surface may be a more suitable method for eDNA sampling in headwaters, as it presents a lower cost and shorter working time than bottom sampling.

In conclusion, we designed the qPCR primer/probe set for *O*. *japonicus* using the 12S rRNA region and successfully detected its distributions using the eDNA method, the results of which we compared with the hand-capture method. The eDNA methods will be useful for endangered species, because we should not disturb their habitats [21]. In addition, in rivers and streams with strong currents, a direct survey can be difficult and dangerous. Utilizing eDNA detection methods with samples collected from the surface of the water to estimate species distribution is a safer alternative method.

## Supporting information

S1 File MIQE check list. (XLS)

S1 Table All data which used in this study. (XLSX)
